# CRISPR Gene Tagging for Illuminating Endogenous Protein Dynamics

**DOI:** 10.3390/ijms27125584

**Published:** 2026-06-20

**Authors:** Nader Afifi, Dennis Colussi, Oscar Perez-Leal

**Affiliations:** Moulder Center for Drug Discovery Research, Department of Pharmaceutical Sciences, School of Pharmacy, Temple University, Philadelphia, PA 19140, USA; nader.afifi@temple.edu (N.A.);

**Keywords:** CRISPR/Cas9, gene tagging, HDR, high-content imaging, knock-in

## Abstract

Endogenous gene tagging using CRISPR has changed the understanding of the role played by different proteins due to the ability to track and study proteins in their natural state. With CRISPR-based gene tagging, it is possible to insert fluorescent, luminescent, epitope, affinity, and proximity labels into the target protein at its endogenous genomic location without affecting its physiological expression and dynamics. Here, we discuss the DNA-repair mechanisms employed in endogenous gene tagging, including homology-dependent repair, NHEJ-based integration, and alternative approaches that can be used with challenging cell types. Key aspects of efficient CRISPR tagging experiments are also described. Additionally, we review recent advances in the increasing array of protein tag technologies, including fluorescent proteins, split-reporter technologies, NanoLuc/HiBiT, peptide epitopes, and proximity biotinylation enzymes. Lastly, we review the scalability of endogenous tagging approaches using multiplex editing, atlas-scale proteome tagging, iPSC-based disease modeling, and drug discovery platforms for assessing target engagement, protein degradation, phenotype screening, and mechanism of action of compounds. Although difficult in primary and pluripotent cells, new methods based on avoiding double-strand breaks, such as prime editing, PASTE, and CRISPR associated transposases, will drive the future expansion of endogenous tagging approaches. Such developments firmly set up CRISPR gene tagging as a fundamental technology in quantitative cell biology and translational pharmacology.

## 1. Introduction

In order to determine the function of a protein, scientists must trace its localization, dynamics, and interactions within living cells. Traditional methods are limited in their ability to do this: recombinant tagged proteins, when artificially over-expressed, can interfere with the endogenous stoichiometry of native proteins and induce artificial localization [1]. Immunofluorescence staining of fixed cells only provides a static image of a process that is naturally dynamic [2].

CRISPR/Cas9-mediated gene editing offers a novel alternative to study proteins under native physiological conditions [3]. A CRISPR endonuclease can be used to generate a gene-specific DNA double-strand break (DSB) and a co-delivered DNA donor template, which directs the incorporation of a fluorescent protein or short epitope tag at the chosen genomic locus while preserving the cell’s normal transcriptional and post-transcriptional regulation of the tagged protein [4]. Thus, endogenously tagged proteins are expressed at physiologically relevant levels and enable live cell imaging and high-resolution sub-cellular protein localization studies [5,6].

Here, we review the underlying DNA-repair mechanisms that support CRISPR/Cas9-mediated gene tagging; the single guide RNA (sgRNA) selection, donor template format, and tag-placement choices that determine experimental success; the strategies that have been developed to improve homology-directed repair (HDR) efficiency; the current capacity of this technology for tagging complex cellular models such as induced pluripotent stem cells (iPSCs); and the approaches available for multiplex gene tagging, which allow several endogenous proteins to be detected simultaneously in the same cell.

Beyond these methodological foundations, we also survey the expanding toolkit of protein tags, including fluorescent, luminescent, epitope, affinity and proximity-labeling tags, and the growing use of endogenously tagged cell lines in proteome-scale atlases, iPSC disease models, and quantitative drug discovery. Unlike existing reviews that treat CRISPR genome editing or fluorescent imaging in isolation, we connect the underlying repair biology and experimental design directly to these downstream translational applications. Throughout, we keep the central limitation of the technology in view: HDR remains inefficient and restricted to dividing cells, so the reliable tagging of post-mitotic, primary, and pluripotent models is still the principal unresolved challenge, and it is this gap that is now motivating a shift toward emerging double-strand-break-free strategies, which we consider as the likely next phase of the field.

## 2. CRISPR/Cas9-Mediated Gene Tagging: Principles and Mechanisms

The CRISPR/Cas9 system, initially identified as an adaptive immune system in bacteria, has been adapted to genome engineering to allow the precise targeting and modification of mammalian DNA [7]. It is the most widely used genome editing platform because of its versatility, simplicity, and programmability. This programmability can be harnessed for endogenous tagging, where a fluorescent reporter or epitope tag sequence can be inserted at a desired genomic locus without affecting native gene function or regulation [8].

**Figure 1:**
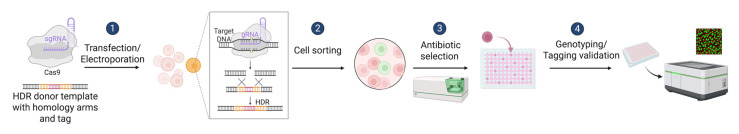
Overview of the workflow of CRISPR/Cas9-mediated endogenous gene tagging and clone generation. (1) Cells are transfected with CRISPR/Cas9 components including an HDR donor template containing the desired tag flanked by homology arms. Cas9 induces a site-specific DSB. HDR then takes place. (2) Cells are enriched and sorted via fluorescence-activated cell sorting (FACS). (3) Antibiotic selection then takes place to isolate cells that were successfully edited. (4) Cells with the right inserted tag are then validated by means of PCR, sequencing, fluorescence imaging, or protein expression analysis.

The accurate, site-specific cleavage of DNA followed by template-directed repair is essential for the ability of CRISPR/Cas9 to tag endogenous genes. An sgRNA directs the Cas endonuclease to a complementary DNA sequence to initiate a DSB, determining the location of the cut (Figure 1). The target sequence must be adjacent to a protospacer adjacent motif (PAM), typically 5′-NGG-3′ for Streptococcus pyogenes Cas9 (SpCas9), the most commonly used Cas9 orthologue in mammalian genome editing; this PAM requirement is what confers specificity for DNA binding and cleavage [7,8]. After cleavage, Cas9 produces blunt-ended DNA fragments via the concerted action of two nuclease domains, HNH and RuvC, cleaving the complementary and non-complementary strands, respectively [9,10].

The resulting DSB is the entry point for endogenous DNA repair, which ultimately determines whether the tag is incorporated. The break should be close to the desired insertion site for efficient tagging, usually at the N- or C-terminus of the coding sequence. After the DSB is formed, the cell uses one of two main repair pathways: non-homologous end joining (NHEJ) and HDR, which differ in their repair mechanism, accuracy, and applicability to endogenous tagging [11,12]. NHEJ ligates the two broken ends without a template, operates throughout the cell cycle, and frequently introduces small insertions or deletions (indels) at the junction [13,14,15]. This imprecision makes NHEJ the route of choice for gene knockout, but unsuitable for inserting an in-frame reporter. HDR is the pathway that makes endogenous gene tagging possible. It operates only during the S and G2 phases of the cell cycle, when the homologous recombination machinery is engaged in repairing breaks at the replication fork using the sister chromatid as a template [15,16]. When a researcher supplies an exogenous donor carrying the desired tag flanked by sequences homologous to the regions on either side of the cut, this same machinery can be redirected to copy the donor sequence, rather than the sister chromatid, into the genomic locus, producing a clean in-frame insertion that is stably propagated to all daughter cells. The architecture of the donor is therefore a critical design parameter: short single-stranded oligodeoxynucleotides (ssODNs) with 35–90 nt homology arms are typically used for epitope or short peptide tags, whereas plasmid or PCR-product donors with 500–1000 bp arms are used for larger inserts such as fluorescent proteins. ssODNs, in particular, have been shown to improve knock-in efficiency in mammalian cells [17]. Although HDR is intrinsically less efficient than NHEJ and is restricted to dividing cells, the precision it offers makes it the dominant strategy for inserting fluorescent and epitope tags at the endogenous locus.

Various alternative knock-in strategies have been devised so that the efficiency of repair via NHEJ can be exploited to efficiently knock-in donor DNA to the cleavage site. For example, homology-independent targeted integration (HITI) [18] does not require homology arms. The donor plasmid contains recognition sequences for Cas9, which flank the insert; the plasmid and genomic DNA are cleaved to give two blunt compatible ends, which the cell then ligates via NHEJ. This strategy is useful for tagging multiple cell types, including non-dividing cells in which HDR is not active, but the overall knock-in efficiency at any particular locus is still low, and the method is best suited for the insertion of a large cassette, rather than the knock-in of SNPs genetic mutations. Furthermore, HITI has been observed to give higher off-target integration rates than HDR strategies, which is also a concern in tagging applications [18,19,20].

Microhomology-dependent targeted integration (MITI) is another approach that uses the microhomology-mediated end joining (MMEJ) pathway, which aligns the free ends of the DNA through microhomologous regions before ligation [21]. For MITI, the nuclease Cas12a is used, which is a class V CRISPR-associated nuclease. Cas12a has very different catalytic mechanisms from Cas9, as it recognizes T-rich (5-TTTV-3) PAM sites and cleaves to form staggered, 5′ overhangs, while Cas9 recognizes G-rich (5-NGG-3) and cleaves blunt ends. Cas12a has lower off target activity and very selective mismatch discrimination compared to Cas9, which makes it well suited to the insertion of a reporter gene [22,23,24]. MITI has been used to insert a tdTomato reporter gene into a constitutively expressed CLTA in 293T cells and into the non-expressed GREB1L gene in fibroblasts, both resulting in approximately four times more precise editing than with HITI [21]. However, like HITI, MITI again relies on a suite of error-prone repair pathways, and small indels may still be generated at the junctions. In summary, HITI and MITI offer additional options for endogenous tagging in post-mitotic cells, but HDR is the preferred approach for accurate gene tagging in mitotic cell lines such as iPSCs, as described below.

## 3. Protein Tags for Endogenous Labeling

**Figure 2:**
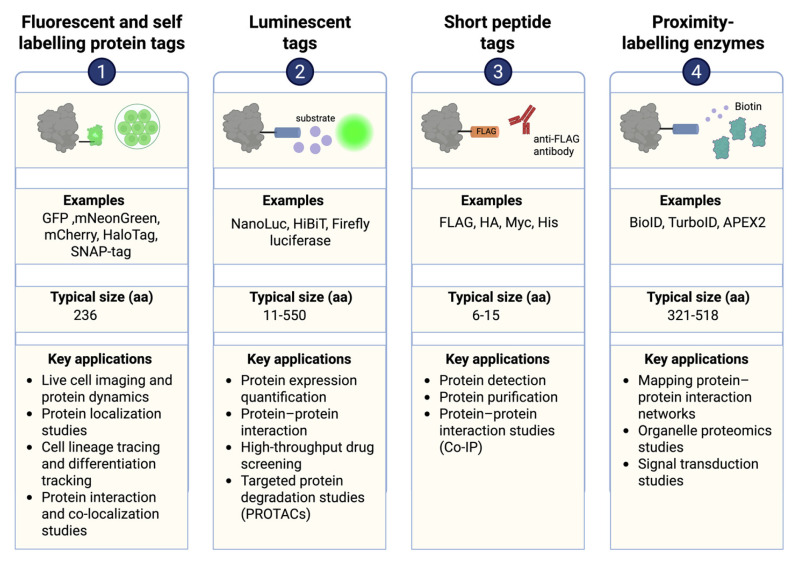
Types of tags used in CRISPR/Cas9-mediated endogenous gene tagging and their major applications.

The tag is chosen according to the experimental purpose since each protein tag gives the tagged protein different properties (Figure 2). The most commonly used tags for CRISPR/Cas9-mediated endogenous tagging can be classified into four broad categories: fluorescent and self-labeling protein tags, mainly for live-cell and high-resolution imaging; luminescent tags, for the quantitative readout of protein abundance and target engagement; short peptide tags, for affinity capture and antibody-based detection; and proximity-labeling enzymes, to map the local protein environment of a tagged bait. The compromise between signal strength, tag size and steric perturbation of the host protein governs which tag is best suited to a given experiment.

### 3.1. Fluorescent Protein Tags

Fluorescent proteins (FPs) are the most used class of tags in CRISPR/Cas9-mediated endogenous labeling, as they allow the real-time imaging of protein localization and dynamics in living cells without the need for exogenous substrates [25]. Classic monomeric FPs such as GFP, mCherry, mScarlet, and mNeonGreen can be introduced to endogenous loci using CRISPR/Cas9-mediated HDR to create knock-in cell lines that express one or more fluorescently tagged proteins. For example, Ratz et al. labeled VIM, ZYX, and HMGA1 with rsEGFP2 via CRISPR, which allowed RESOLFT super-resolution imaging at physiological expression levels [26]. The same logic has been used to trace protein redistribution under perturbation, such as the spatial response of metabolic enzymes to oxidative stress [27]. The major limitation of fluorescent tags is steric. A fully folded FP adds 25–30 kDa to the host protein and can interfere with folding, localization, or function [28]. Thus, the choice of tags is a compromise between the brightness and photostability of the FP and the size and folding kinetics of the FP in relation to the host protein.

### 3.2. Split Fluorescent Proteins

To reduce this steric burden, split-FP strategies divide the fluorescent protein between the host protein and a separately expressed complement. In the split-GFP system, a small β-strand (usually GFP11, ~16 aa) is fused to the endogenous protein, and the larger GFP1-10 fragment is provided in trans [29]. Because HDR requires the insertion of only a small peptide, the efficiency of knock-in is significantly higher than for full-length FPs, which allowed the scalable tagging of >1000 human protein-coding genes in the OpenCell project [30]. The trade-off is that the assay requires reliable expression of the complementary fragment in the same cell, adding a level of system complexity not present with full-length FPs.

### 3.3. Self-Labeling Protein Tags

A complementary group of tags works by binding a small fluorescent molecule rather than by directly emitting fluorescence. HaloTag (~33 kDa), SNAP-tag, and CLIP-tag covalently react with synthetic ligands carrying any desired fluorophore, biotin, or affinity handle [31]. Being inserted at the endogenous locus, the tags allow the same cell line to be imaged in different colors, pulse chased with different dyes, or captured for downstream biochemistry depending on the ligand provided. They are particularly popular for super-resolution and single-molecule imaging, since they can be combined with the bright, photostable Janelia Fluor (JF) dye family [32], which often performs better than genetically encoded FPs at high spatial resolution.

### 3.4. Luminescent Tags

Instead of fluorescence excitation, luminescent tags produce light via the enzymatic oxidation of a small molecule substrate, resulting in an extraordinarily low cellular background and very high signal-to-noise. Firefly luciferase (~62 kDa) and Renilla luciferase (~36 kDa) have historically been the workhorses of the field, but their size makes them less attractive for CRISPR knock-in. NanoLuc (~19 kDa) is a luciferase from the deep-sea shrimp *Oplophorus gracilirostris* [33,34,35,36] that is ~150-fold [33] brighter per molecule than firefly luciferase and is small enough to be tolerated as a C- or N-terminal fusion at most endogenous loci. Its split derivative HiBiT is an 11-amino acid peptide that complements the larger LgBiT fragment in trans to restore full luciferase activity [37]. Because HiBiT only requires a short peptide to be inserted via HDR, knock-in efficiency is high and CRISPR-engineered HiBiT cell lines have been used to measure endogenous protein abundance with high signal to noise, monitor protein degradation in PROTAC and molecular-glue screens, and measure target engagement in cellular thermal shift assays (CETSAs) [38,39]. The main drawback of luminescent tags is that they require an exogenous substrate (furimazine for NanoLuc and HiBiT) and offer a lower spatial resolution than fluorescent reporters. They are thus more suited for plate-based, kinetic, and population-level readouts rather than subcellular imaging.

### 3.5. Short Peptide Tags

Short peptide tags such as FLAG, HA, Myc, V5, His-tag, and Strep-tag are usually less than 15 amino acids and have a dual purpose, serving as affinity handles for purification and immobilization, as well as epitopes for antibody-based detection [40,41]. Often the same tag is used for both purposes; FLAG and HA for example are routinely used both for immunoprecipitation and for Western blotting from the same construct. The distinction between ‘affinity’ and ‘epitope’ tags is therefore largely an operational one. These tags, when introduced through CRISPR at the endogenous locus, allow for purification and detection of the protein at its native abundance and stoichiometry, avoiding the variable specificity of antibodies raised against the endogenous protein itself [27]. This strategy is especially useful for transcription factors, chromatin proteins, and other targets that do not possess validated antibodies and is the basis of methods such as CRISPR epitope tagging ChIP-seq (CETCh-seq) where an epitope is introduced at the C-terminus of a transcription factor and the locus subsequently identified genome-wide via ChIP-seq using a universal antibody against the tag [42,43].

### 3.6. Proximity-Labeling Tags

Engineered enzymes such as BioID, TurboID [44], and APEX2 covalently biotinylate any biomolecule within a few nanometers of the tagged bait, creating a snapshot of the local interactome that can then be enriched via streptavidin pull-down and identified through mass spectrometry. CRISPR tagging of bait at its endogenous locus provides a proximity profile indicative of native expression and stoichiometry, as opposed to the artefactual interactions often associated with overexpression of a transfected fusion construct [45,46]. TurboID is especially rapid (10–30 min labeling reactions) and active in a broad range of cell types and tissues, which makes it well suited to capture transient or weak interactions in physiologically relevant contexts [47]. APEX2 requires hydrogen peroxide (H_2_O_2_) to initiate biotinylation, which can cause cytotoxicity due to oxidative stress from H_2_O_2_ beside the high background from non-specific biotinylation of the proteins that are not in the vicinity of the protein of interest [48].

## 4. Design Considerations for CRISPR/Cas9-Mediated Endogenous Tagging

**Figure 3:**
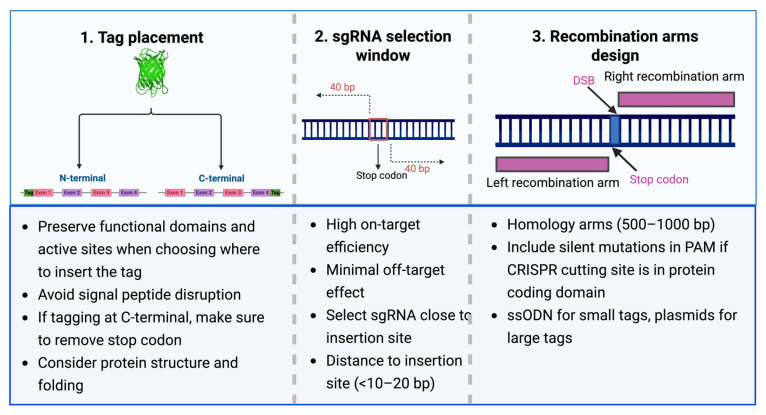
Key design considerations for successful CRISPR/Cas9-mediated endogenous gene tagging. The top panel presents a schematic representation of major design parameters involved in endogenous tagging, including tag placement, sgRNA selection, and recombination arm design. The bottom panel highlights important factors that influence editing efficiency and the preservation of endogenous protein function.

The success of an endogenous tagging experiment depends on three interrelated design decisions, including the sgRNA used to generate the DSB, the donor template that carries the tag into the locus, and the location of the tag within the host protein (Figure 3). Each needs to be optimized in the context of the other, as a perfectly placed tag is useless if the CRISPR cut is inefficient, and a high-efficiency cut at the wrong position can disrupt the function of the protein under study.

### 4.1. sgRNA Selection

The choice of the sgRNA site is the key factor for successful CRISPR/Cas9-mediated gene tagging because it determines the efficiency of cleavage at the target locus, in addition to preventing unwanted cuts at the rest of the genome [49,50]. Unlike generic CRISPR genome editing to generate knockouts, where any sgRNA that disrupts the gene is sufficient, sgRNA selection for endogenous tagging is limited by the position of the desired insertion site, generally close to the start (for N-terminus tagging) or stop codon (for C-terminus tagging) of the coding sequence. Thus, the selection of the target site represents a real experimental limitation. First of all, a PAM has to be found near the desired cleavage site, preferably within 10–20 bp, so that the DSB is created close enough to the insertion site to be repaired via HDR with the help of the donor template [51].

In addition to positional constraints, the sgRNA sequence composition affects on-target activity and off-target risk. A GC content of ~40–60% helps to form stable sgRNA–DNA hybridization, while limiting the formation of alternative secondary structures [50,52,53]. The on-target efficiency and the off-target activity must always be balanced together: a guide with maximal cleavage activity is of little value if it also cleaves elsewhere in the genome. Several modifications in the sgRNA have been developed to reduce off-target activity, including chemical modification of the sgRNA backbone [54] and changes to the sgRNA length and composition [54]. Truncated guides of 17–18 nt have been shown to reduce off-target activity, without compromising on-target precision [55,56]. The GGX20 strategy adds two guanines at the 5′ end of a standard 20 nt spacer, which improves specificity via a decreased tolerance for mismatches in the seed region [57,58]. Computational tools such as CHOPCHOP v4 (https://chopchop.cbu.uib.no/ (accessed on 10 June 2026)) and Benchling designer (https://www.benchling.com/crispr (accessed on 10 June 2026)) rank the candidate sgRNA based on predicted on-target activity, the GC content, and potential off-target sites [50,59,60], and dedicated off-target prediction tools such as Cas-OFFinder, CCTop, and CRISPRscan scan the genome for sequences with partial complementarity to the sgRNA. In the case of unwanted transcript effects, the cleavage site should also be selected to avoid intronic or exonic splice sites.

At the same time, engineered Cas9 variants have been developed to directly enhance cleavage fidelity. High-fidelity orthologues such as SpCas9-HF1 [61], eSpCas9(1.1) [62], and HypaCas9 [63,64] have amino acid substitutions in the REC3 and HNH domains that destabilize binding to mismatched target sequences and hence reduce off-target cleavage. Since HDR-mediated insertion relies on efficient cleavage and precise repair, using a high-fidelity Cas9 and selecting the most suitable sgRNA results in an increased ratio of accurately tagged knock-in clones.

### 4.2. Design of Donor Template

The choice of donor template depends on the size of the tag to insert, the type of cell, and the experimental constraints. ssODNs are the preferred format for small insertions such as short peptide or epitope tags due to their high HDR efficiency, low cost of synthesis, and tolerance of short (35–90 nt) homology arms [65,66]. In ssODN donor design to enhance HDR efficiency, Richardon et al. showed that asymmetric ssODNs designed with longer homology on the PAM-distal side of the Cas9 cleavage site and shorter homology on the PAM-proximal side significantly improved HDR-mediated genome editing compared with symmetric donor designs [67]. Double-stranded donors are required for larger insertions, e.g., full-length fluorescent proteins (∼720 bp for GFP). Precise integration at the target locus is facilitated by plasmid donors containing the tag flanked by 500–1000 bp homology arms [68] and can accommodate more complex designs, including selection markers [69] and additional regulatory elements, although their knock-in efficiency is generally lower than that of ssODNs. For cell types or tissues that are difficult to transfect, including iPSCs, viral vectors such as adeno-associated virus (AAV) or lentivirus can deliver the donor with much higher efficiency, but with additional concerns of immunogenicity, off-target effects and, for integrating viruses, the risk of insertional mutagenesis [70,71].

### 4.3. Tag Placement Design

Tags can be added to the N- or C-terminus of the host protein. C-terminal tagging is more often chosen because the N-terminus can contain important targeting information (signal peptides for the secretory pathway, mitochondrial targeting sequences, etc.) and is often the target of co-translational modifications such as initiator-methionine excision and N-terminal acetylation, any of which could be disrupted by an N-terminal tag [72,73]. N-terminal tagging becomes a preferred location if the C-terminus itself contains function-critical features such as CAAX prenylation motifs (e.g., small GTPases), GPI-anchor signals, PDZ-binding sequences, or post-translational cleavage that will remove the tag; in these cases, the tag is put downstream of the initiator methionine or any cleaved signal peptide so that the mature protein retains its native C-terminus. For C-terminal tagging, the stop codon of the target gene needs to be removed from the HDR donor template to allow translation to proceed into the tag to generate a single contiguous fusion protein [74,75]. For proteins where both termini are functionally important, an internal tagging site can be engineered with an appropriate design of flexible linker sequences between the tag and the host protein in order to maintain folding and activity [76,77]. Alternatively, the CRISPR/Cas9-mediated insertion of exon (CRISPIE) technique consists of inserting a synthetic exon encoding a fluorescent reporter into an intron of the host gene, allowing high-fidelity and reversible labeling of the protein with minimal perturbation to the native gene structure [30,78]. Instead, if a stable physical fusion is not required, the Porcine teschovirus-1 2A (P2A) peptide-mediated ribosomal skipping can be inserted between the endogenous protein and the reporter. Ribosomal skipping at the P2A sequence generates two independent polypeptides from a single open reading frame, leaving the host protein untagged while co-expressing the reporter or selectable marker in stoichiometric proportion [79].

### 4.4. Standardization and Scalability

Although each tagging experiment should be optimized for the specific gene and cell type, the same design principles can be applied on a larger scale. For instance, the OpenCell collection was produced by the combination of standardized donor architecture, consistent C-terminal tag placement, and standardized clonal-validation pipelines for the integration of split fluorescent tags into more than a thousand human endogenous loci in one cell line [30]. The success of such large-scale efforts demonstrates that the design choices described in this section are not just per-experiment heuristics but scale, with proper automation, to library-level operations.

## 5. Strategies to Improve Gene Tagging Efficiency

HDR remains the rate-limiting step in CRISPR/Cas9-mediated endogenous gene tagging, particularly in cell types that are intrinsically poor substrates for editing, such as iPSCs. Several complementary strategies have been developed to bias repair towards HDR and away from NHEJ, which can be broadly divided into three categories: (i) small-molecule inhibition of NHEJ to modulate DNA repair pathway choice; (ii) cell-cycle synchronization to enrich the population in S and G2 phases where HDR is active; and (iii) optimization of donor template design for selecting edited cells.

### 5.1. Inhibition of NHEJ

Since NHEJ is the major repair pathway in mammalian cells and directly competes with HDR on the same broken ends, it is therefore logical to decrease the activity of NHEJ to increase the proportion of breaks that are channeled through HDR [66,80]. For this, cell-permeable small-molecule inhibitors of NHEJ have been developed [81,82]. One major class targets the catalytic subunit of DNA-dependent protein kinase (DNA-PKcs), an early and essential component of the NHEJ machinery [83]. DNA-PKcs inhibitors, such as NU7026, NU7441, KU-0060648, and M3814, all result in a significant increase in HDR-mediated knock-in efficiency in a variety of cell types [84,85,86]. A second class targets DNA Ligase IV, the enzyme that seals broken ends in NHEJ [87,88]. SCR7, the best-characterized Ligase IV inhibitor, binds the LIG4 DNA-binding domain and prevents formation of the LIG4/XRCC4 complex that is essential for ligase stability and activity [89] and has been shown to increase HDR-mediated knock-in by around four-fold in HEK293 cells [90].

### 5.2. Cell-Cycle Synchronization

Since HDR is limited to the S and G2 phases of the cell cycle, enriching the cell population in these phases offers a complementary route to increased knock-in efficiency [91]. Synchronization can be achieved through serum starvation or by using chemical inhibitors of cell-cycle progression [92,93]. Nocodazole, a microtubule-depolymerizing agent that arrests cells at the G2/M boundary, increases the efficiency of HDR-mediated tagging by approximately two-fold [94,95]. Aphidicolin, a DNA polymerase α inhibitor, blocks cells in early S phase, while ABT-751, a microtubule-disrupting agent, blocks cells at G2/M, with comparable enrichments in different cell lines including pluripotent stem cells [92,96]. Other small-molecule approaches that act on the repair-pathway balance in a more general way are histone deacetylase inhibitors (HDACis), 53BP1 inhibitors, and Ku70/Ku80 heterodimer inhibitors [97,98]. While these strategies can reproducibly improve HDR, they have limitations that are relevant for endogenous tagging: most require further optimization to reduce cytotoxicity and improve stability in the target cell type, and the magnitude of their effect varies considerably between cell lines [99,100], which is particularly problematic in sensitive cell types such as iPSCs.

### 5.3. Donor Template Optimization

A third way to increase tagging efficiency targets the donor template rather than the cell. Recent advances have led to the development of modular donor-backbone systems that simplify the assembly of HDR templates for many target loci, such as the FAST-HDR system [101]. The FAST-HDR empty vector is built around a labeling-tag module fused to a mammalian antibiotic selection cassette, which is only active after successful in-frame integration at the endogenous locus, so that cells surviving antibiotic selection are correctly tagged knock-in clones rather than cells with random insertions. A functional, gene-specific donor can be generated with only one Gibson assembly reaction to insert the two gene-specific homology arms into the empty backbone, and the process can be completed in about one day. Co-transfection of this donor with a plasmid encoding the high-fidelity nuclease eSpCas9(1.1) and the matching gene-specific sgRNA then enables gene tagging followed by the rapid selection of endogenously tagged pool cell lines, in contrast to traditional cloning workflows that require several weeks to assemble a custom donor and generate a tagged cell line [77,102]. Beyond FAST-HDR, qTAG is another example of a plasmid scaffold that supports both N-terminal and C-terminal endogenous gene tagging for the targeting of both HDR and MMEJ pathways. The system incorporates multiple tag configurations and selectable-marker customization by means of the strategic restriction sites within the cassettes that allows the flexible exchange of tagging cassettes [46].

### 5.4. Cas9 RNP Delivery Combined with Single-Stranded DNA Donor Templates

A fourth strategy for improving tagging efficiency concerns the physical form in which Cas9 and the HDR donor are delivered. When Cas9 is expressed from a plasmid, the nuclease accumulates over hours to days, prolonging DSB formation and increasing off-target cleavage. The delivery of Cas9 pre-assembled with its guide RNA as a ribonucleoprotein (RNP) complex instead introduces a transient pulse of nuclease activity: the protein is rapidly degraded by the proteasome, sharply reducing off-target editing and DSB-associated toxicity without compromising on-target efficiency [103]. Off-target rates are 2- to 10-fold lower across multiple guide RNAs compared to plasmid-delivered Cas9, and in iPSCs, which are especially sensitive to prolonged Cas9 expression, RNP electroporation has been shown to be a useful strategy for endogenous protein tagging [103,104].

The optimal donor template format depends on the tag size. For short insertions such as epitope tags (FLAG, HA, HiBiT), ssODNs of 120–200 bp with symmetric homology arms of 30–60 nucleotides flanking the insertion site are sufficient, carry no risk of random genomic integration, and achieve HDR efficiencies up to 60% in cell lines under optimized conditions [105,106]. For full-length fluorescent proteins or self-labeling enzyme domains (700–900 bp), long single-stranded DNA (lssDNA) templates or modified double-stranded donor blocks with homology arms of 200–300 bp are required [80]. The brief activity window of RNP is particularly advantageous in animal embryos: the microinjection of Cas9 RNP with asymmetric ssDNA repair templates into one-cell-stage zebrafish embryos yielded the germline transmission of epitope tag knock-ins at rates of 17–29% and correct knock-in allele frequencies of 3–8% across multiple endogenous loci, among the highest reported without AAV or selection in any vertebrate model [107,108,109]. In immunologically sensitive primary cells such as T lymphocytes and hematopoietic stem and progenitor cells, RNP electroporation has enabled HDR-mediated fluorescent and epitope tag knock-in at endogenous loci with efficiencies approaching 20%, outcomes not achievable through viral or plasmid delivery [110]. Matching RNP delivery to an appropriately formatted donor template therefore represents a broadly applicable enhancement to standard CRISPR/Cas9-HDR tagging workflows across dividing cell lines, iPSCs, primary immune cells, and animal embryos.

## 6. Multiplex Gene Tagging

Most cellular processes are carried out by groups of proteins acting in concert, and single-tag experiments capture only one component at a time. Signal transduction cascades, organelle dynamics, and cell growth all depend on the spatiotemporal coordination of multiple proteins [111,112,113], which is difficult to follow with a single endogenous reporter. Multiplex tagging, defined as the simultaneous labeling of two or more endogenous proteins in the same cell, addresses this limitation and is increasingly used to follow the dynamics of multi-protein processes in live cells, using techniques such as high-content imaging.

In practical terms, multiplex tagging is achieved by co-delivering several sgRNAs together with their corresponding donor templates, with each sgRNA-donor pair targeting a distinct genomic locus to insert a spectrally distinguishable reporter in the same cell. The FAST-HDR system [101], introduced in Section 5, is a representative example: its series of backbone plasmids allows the assembly of donors carrying different labeling tags, so that multiple fluorescent proteins can be inserted in parallel using compatible cloning and selection workflows. FAST-HDR has been used to tag up to three endogenous proteins simultaneously, allowing the high-content imaging of processes such as autophagy and mitochondrial dynamics in cells expressing SARS-CoV-2 viral proteins [101].

The principal limitation of multiplex tagging is that the efficiencies of editing at each locus are compounding rather than additive: because HDR at each locus is an independent event, the probability of obtaining a clone correctly tagged at all loci falls multiplicatively with the number of targets. The FAST-HDR system mitigates this by pairing each tagging event with a different mammalian antibiotic-resistance cassette, so that selecting with all of the corresponding antibiotics together enriches for cells that have undergone every intended insertion. Using this approach, at least 98% of cells surviving the combined selection carry all of the intended tags [101].

## 7. Use of Endogenous Gene Tagging Applications in Molecular Biology and Drug Discovery

**Figure 4:**
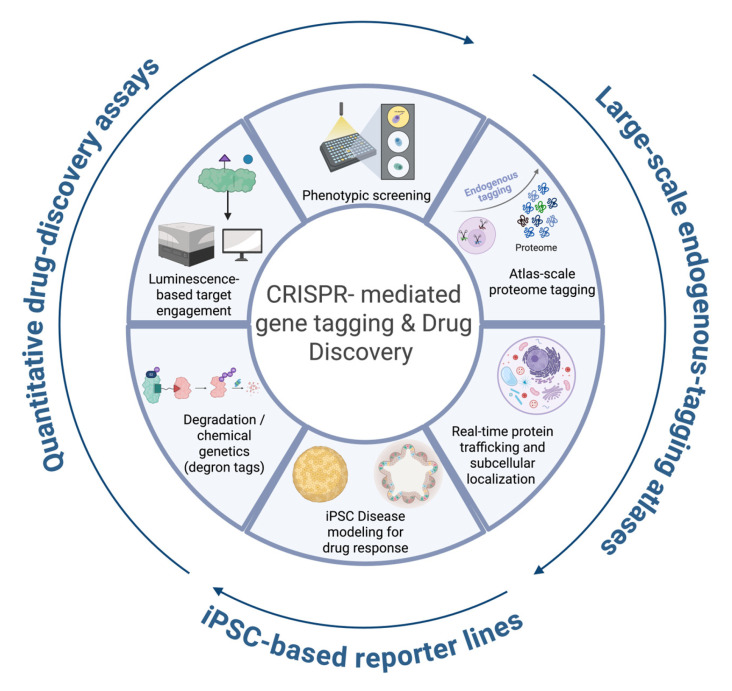
A schematic representation of the three main specific areas using CRISPR/Cas9-mediated gene tagging for applications in molecular biology and drug discovery.

The methods discussed in Section 2, Section 3, Section 4, Section 5 and Section 6 have revolutionized CRISPRCas9-mediated endogenous tagging from a proof-of-principle technology to a routine enabling platform for cell biology, disease modeling, and translational research. The field has been defined by three specific areas of application: large-scale endogenous-tagging atlases mapping the human proteome at native expression, iPSC-based reporter lines that carry the tag through differentiation into disease-relevant cell types, and quantitative drug-discovery assays (Figure 4).

### 7.1. Large-Scale Endogenous-Tagging Atlases

Two community resources demonstrate what is possible when CRISPR/Cas9-mediated gene tagging is done at scale. The OpenCell project tagged 1310 human protein-coding genes endogenously in HEK293T cells with split-fluorescent-protein cassettes and combined the resulting imaging with mass spectrometry interactome data and machine-learning analysis to produce a unified atlas of subcellular localization and protein interaction networks at near-native expression [30]. The Allen Institute for Cell Science has generated a parallel set of endogenously tagged human iPSC lines that currently span 61 structural proteins across all major organelles, the cytoskeleton, and nuclear architecture [114]. Because the parental cell type is pluripotent, the same tagged line can be differentiated into any lineage needed. Together, these efforts demonstrate that endogenous tagging can be parallelized, standardized, and made publicly available and that the resulting cell lines are reusable platforms for cell biology, rather than one-off experimental constructs.

### 7.2. Endogenous Tagging in iPSCs and Their Differentiated Derivatives

iPSCs are considered a difficult cellular model for CRISPR gene tagging due to the following challenges: HDR efficiency is typically <1% without selection, and iPSCs are especially sensitive to DNA damage and apoptosis induced by CRISPR/Cas9-mediated double-strand breaks [102,115,116,117]. However, the successful development of a tagged iPSC cell line is highly rewarding because the engineered tag is permanently incorporated into the genome and is passed on to all daughter cells upon differentiation. Thus, a tagged iPSC clone can be differentiated into common cell types such as neurons, cardiomyocytes, hepatocytes, hematopoietic cells, or three-dimensional organoids, and the tag is continuously expressed under the control of the endogenous promoter [118]. It is also possible to tag genes that are silent in the pluripotent state and expressed only after differentiation [114].

The utility of tagging iPSCs is illustrated by several recent examples. Husser et al. tagged actin, α-tubulin, RhoA, and anillin at endogenous loci in human iPSCs with split-mNeonGreen and thus enabled live-cell imaging and the quantitative analysis of contractile-ring assembly and cytokinetic furrow ingression at native protein levels [119]. CRISPR-engineered iPSC lines with the HA epitope or mCherry knocked-in at the endogenous SNCA locus retained normal electrophysiological properties after differentiation to neurons and enabled the direct visualization of α-synuclein accumulation under lysosomal stress, thus providing a tractable system for the study of Parkinson’s disease and related synucleinopathies [120]. In the heart field, the endogenous tagging of the α-myosin heavy chain gene MYH6 with EGFP generated iPSC-derived cardiomyocyte lines that can be tracked through differentiation [121], while a parallel MYH6-mScarlet knock-in allows continuous live-cell monitoring in both two- and three-dimensional cardiomyocyte cultures for cardiotoxicity testing, biomaterial evaluations, and functional phenotyping [122]. Tagged iPSC derivatives also enable lineage tracing and the FACS-based isolation of differentiated populations, which has been used to obtain highly enriched cell populations for downstream molecular and functional analyses [123]. Taken together, these studies demonstrate the versatility of CRISPR/Cas9-mediated iPSC tagging as a tool for investigating protein localization, abundance, and function during differentiation and disease modeling in physiologically relevant human cell models.

### 7.3. Drug Discovery and Target-Engagement Applications

Drug discovery needs assays that report the cellular pharmacology of a compound at the protein abundance and cellular context that the drug will actually encounter. Endogenous tagging has therefore been widely adopted across pharmacological workflows, because it allows the measurement of protein abundance, localization, and dynamics at native expression and stoichiometry rather than at the artefactually elevated levels generated through transgenic overexpression. Four application paradigms now dominate the space: phenotypic drug screening with fluorescently tagged organelle and structural reporters; luminescence-based target-engagement and degradation assays; chemical-genetic target validation via degron-tagged knock-in; and in vitro disease modeling.

#### 7.3.1. Phenotypic Drug Screening with Endogenously Tagged Reporters

Live-cell, multiplex gene-tagged cell lines generated via CRISPR/Cas9-mediated endogenous tagging are very useful for drug screening. Recently, we developed a HeLa cell line with mClover3-tagged β-tubulin and mTagBFP2-tagged histone H1 using the FAST-HDR system, where we screened a 429-compound kinase-inhibitor library in 384-well format through high-content imaging to identify inhibitors of tubulin polymerization in live cells [124]. Haralick texture analysis of the live-cell microtubule signal identified three tubulin polymerization inhibitors (ON-01910, HMN-214, and KX2-391), and time-lapse imaging confirmed the rapid depolymerization kinetics, all without cell fixation or immunostaining. On a larger scale, Cox and colleagues at Janssen developed a panel of 15 reporter cell lines encompassing 12 endogenously tagged organelle markers and pathway effectors across three lineages and used it to phenotypically profile 1008 approved drugs and tool compounds, each at four concentrations. The resulting live-cell imaging fingerprints correctly ranked compound mechanism-of-action for 41 out of 83 testable mechanism of action (MoA) categories at an AUC-ROC ≥ 0.9, demonstrating that panels of endogenously tagged organelle reporters can be used as general-purpose MoA classifiers [125].

#### 7.3.2. Luminescence-Based Target Engagement, Degradation, and GPCR Pharmacology

HiBiT- and NanoLuc-tagged endogenous cell lines serve as a workhorse in quantitative drug discovery. The first demonstration of CRISPR/Cas9 insertion of the 11-amino-acid HiBiT peptide at endogenous loci [37] was quickly followed by a high-throughput workflow with ∼86% insertion success across diverse targets and cell lines, with better preservation of native biology than overexpression systems [6]. Riching and colleagues have implemented a high-throughput protocol for targeted protein degradation that uses HiBiT knock-in cell lines to profile PROTAC and molecular-glue compounds in either kinetic live-cell or endpoint lytic format, measuring the DC50, Dmax, and degradation rate [126]. The approach is now supported by libraries of hundreds of pre-built HiBiT knock-in clones for targets including AKT1, BRD4, KRAS, and many GPCRs, and a published RNP-delivery protocol brings the workflow within reach of standard drug-discovery laboratories [127]. Similar luminescent chemistry has been adapted for plate-based target engagement using the HiBiT thermal shift assay (BiTSA), which distinguishes small molecules that target otherwise difficult-to-drug proteins such as mutant KRAS and TP53 [128]. HiBiT-tagged GPCRs have been used in receptor pharmacology for equilibrium and real-time studies of receptor internalization and ligand engagement at endogenous expression, including bioluminescence resonance energy transfer (BRET)-based competitive binding with fluorescent tracers [129]. CRISPR/Cas9-mediated endogenous tagging was used to study post-translational modifications by combining endogenous HiBiT-tagged proteins with fluorescent antibodies that recognize specific protein modifications. This approach enables the sensitive and homogeneous detection of PTMs without the need for protein overexpression or cell lysis [130].

#### 7.3.3. Chemical-Genetic Target Validation with Degron-Tagged Knock-In

Along with reporter readouts, CRISPR gene tagging can be used to insert conditional degron domains into endogenous loci to allow rapid and reversible depletion of the tagged protein on demand. The dTAG system involves fusing an FKBP12-F36V-degron to an endogenous protein via CRISPR/Cas9-mediated gene editing tagging. The fusion product is degraded within minutes after adding cell-permeable dTAG ligands. This platform has been used to validate cancer drug targets such as BRD2, BRD3, BRD4, BRD9, ERRα, RIPK2, and FKBP12 itself and has become a standard chemical–genetic tool to differentiate on-target from off-target effects of small molecules and to assess whether a target is amenable to therapeutic degradation prior to embarking on a full PROTAC discovery campaign [131,132].

#### 7.3.4. Disease-Specific Endogenously Tagged Drug Discovery Models

The use of endogenous protein tags for compound screening in patient-relevant cellular models is highly promising. Recently, Hendriks et al. derived human hepatocyte organoids with CRISPR gene tagging of the lipid droplet marker PLIN2 and created the FatTracer screening platform. They were able to study 35 lipid-metabolism genes and identified FADS2 as a therapeutic target for hepatic steatosis [133]. CRISPR gene tagging was also used for pharmacological phenotyping in patient-mimetic backgrounds with the development of endogenously tagged isogenic iPSC lines carrying Parkinson’s-associated variants of α-synuclein [120] and the Allen Cell Disease Catalog lines carrying cardiomyopathy (MYH7), laminopathy (LMNA), and skeletal-myopathy (MYH3) mutations based on tagged structural-protein backgrounds.

CRISPR/Cas9-mediated endogenous gene tagging now covers most of the drug-discovery pipeline, from chemical–genetic target validation and biochemical purification to phenotypic and mechanism-of-action screening to disease-specific cellular models. Together, these provide a unifying methodology that yields native-pharmacology readouts at each stage.

## 8. Emerging DSB-Free Alternatives for Endogenous Gene Tagging

The CRISPR/Cas9-HDR methodology described in Section 2, Section 3, Section 4, Section 5, Section 6 and Section 7 remains the reference standard for endogenous protein tagging in mammalian cells. Nevertheless, the intrinsic coupling of HDR to the S/G2 phases of the cell cycle, the competing NHEJ pathway that deposits indels on the unedited allele, and the genotoxic stress imposed by the DSB itself present practical constraints, particularly when tagging primary cells or post-mitotic tissues [134]. A second generation of programmable genome-editing technologies that entirely avoid the DNA double-strand break has begun to be applied to endogenous protein tagging, and three systems deserve particular consideration in this context: prime editing, PASTE/PASSIGE, and the recently described evoCAST.

**Prime editing** uses a Cas9 H840A nickase fused to an engineered reverse transcriptase and is directed by a prime editing guide RNA (pegRNA) that carries the desired edit encoded within its own 3′ extension [134]. Because it relies on reverse transcription rather than HDR, prime editing is active across the cell cycle and imposes no DSB, and it produces approximately 4-fold fewer unwanted indels at the targeted locus than conventional Cas9-HDR [134]. For endogenous gene tagging, the central practical constraint is cargo size: the insert sequence must be encoded entirely within the pegRNA 3′ extension, and editing efficiency degrades substantially as the insert length increases beyond approximately 100–200 nucleotides. This means that prime editing is well suited for the insertion of short epitope tags such as FLAG, HA, V5, or HiBiT (8–27 nucleotides) but is not capable of directly inserting full-length fluorescent proteins such as GFP or mScarlet, which encode sequences of approximately 720 nucleotides [134,135]. Prime editing has been used to insert short split-fluorescent-protein peptides to get around this size restriction, most notably the 66-nucleotide mNG2(11) fragment of split mNeonGreen [136]. When expressed in cells stably carrying the complementary large fragment mNG3K (1–10), the inserted short peptide reconstitutes a functional fluorophore, enabling live-cell imaging of the endogenously tagged protein at its native expression level without any DSB or exogenous donor DNA. Building on this, Sanchez et al. [137] created a library of 17,280 pegRNAs targeting 60 endogenous proteins in all subcellular compartments and demonstrated that split-mNeonGreen protein tagging can be accomplished through one single lentiviral transduction event in a pooled fashion with a tagging efficiency directly correlated with chromatin accessibility and gene transcription. By combining the optical readout, in situ sequencing of pegRNAs, and deep learning image analysis, they developed an experimental system for the proteome-wide analysis of localization dynamics of proteins, something currently not possible through HDR-based systems [137]. Twin prime editing (twinPE) extends prime editing in terms of being able to introduce a recombinase landing site through the use of two pegRNAs, which can subsequently be used to introduce a cargo via site-specific recombination, hence providing the ability to insert larger sequences (up to 5 kb) without causing DSBs, thus allowing the insertion of complete fluorescent tags (or any large domain) in one step [138].

**PASTE (Programmable Addition via Site-specific Targeting Elements)** builds directly on this two-step logic by co-delivering, in a single reaction, a prime editor fused to a serine integrase (Bxb1) together with an attB-encoding pegRNA and a large donor plasmid flanked by an attP site [139]. The prime editor installs the integrase-attachment site, and the Bxb1 integrase then catalyzes unidirectional recombination to integrate the donor cargo without generating a DSB. PASTE achieved 10–50% integration efficiency across multiple human cell lines and retained detectable activity in quiescent primary human hepatocytes (~4–5%), demonstrating cell-cycle independence that HDR cannot match [139]. Explicit protocols for EGFP integration in-frame at the endogenous ACTB and NOLC1 loci have been published, confirming the direct applicability of PASTE to fluorescent endogenous tagging [140]. An important limitation is that the Bxb1 recombination event leaves short attL and attR hybrid sequences (~50 bp) at both junctions of the integrated cargo; when the cargo is inserted in-frame with an endogenous open reading frame, these sequences must be engineered to encode an innocuous linker rather than a disruptive peptide [139]. A subsequent iteration, PASSIGE (Prime editing-Assisted Site-Specific Integrase Gene Editing), decouples the prime editor and integrase into separate proteins, removing the steric constraint that limits the fused PASTE architecture. Using recombinase variants evolved via phage-assisted continuous evolution, PASSIGE achieved average targeted gene integration efficiencies of 23% in single-transfection experiments and exceeded 30% at multiple loci in primary human fibroblasts, outperforming PASTE by 9- to 16-fold and representing the most efficient RNA-programmed, DSB-free large-cargo integration reported in primary human cells to date [141].

**evoCAST** is the most mechanistically different of the three approaches. CRISPR-associated transposases (CASTs) are bacterial multi-protein complexes capable of targeting transposable elements to CRISPR-specified genomic sites in a single step without introducing DSBs, using a cut-and-paste transposition mechanism that leave no detectable indels at the target locus [142]. Wild-type CASTs showed negligible activity in human cells, but Witte et al. [142] applied phage-assisted continuous evolution to the TnsB transposase subunit and combined the resulting variant with rationally engineered CAST components to yield evoCAST, which achieved 10–30% integration efficiency across 14 diverse genomic target sites in human cells, representing a more than 420-fold improvement over the wild-type system, while supporting cargo sizes exceeding 10 kb in a single delivery reaction [142]. Compared with PASSIGE, evoCAST offers higher editing purity with essentially zero indels and a simpler single-step integration mechanism that does not require a preceding landing pad installation; however, at present, its overall efficiency is lower, and the system has not yet been systematically evaluated for in-frame endogenous protein tagging across diverse loci or in iPSC-based cellular models [142].

From the perspective of endogenous protein tagging for cell biology, these three technologies complement rather than replace CRISPR/Cas9-HDR. Prime editing with split-fluorescent-protein strategies is currently the most mature DSB-free option for applications requiring the high-throughput, pooled tagging of many proteins simultaneously or the scarless insertion of small functional tags such as epitopes, self-labeling peptide sequences, or degron domains in dividing cell lines and organoids, where the absence of a DSB eliminates indel contamination on the second allele and bypasses the need for clonal selection. PASTE/PASSIGE fills the niche where a full-length fluorescent protein, or a self-labeling enzyme domain such as HaloTag, must be inserted at an endogenous locus in a primary or non-dividing cell type where HDR would be prohibitively inefficient; the att-site scar and the multi-component delivery burden are the main practical trade-offs relative to HDR in that context. evoCAST, with its single-step, indel-free integration and large-cargo capacity, is the most promising of the emerging tools for future high-fidelity tagging in therapeutically and biologically relevant primary cell types, but it awaits broader validation specifically in endogenous tagging workflows before it can be adopted as a routine platform. Until that validation is complete, CRISPR/Cas9-HDR, supported by the optimization strategies described in the preceding sections, remains the method of choice for the generation of endogenously tagged lines intended for quantitative cell biology, disease modeling, and drug discovery.

## 9. Conclusions

CRISPR/Cas9-mediated endogenous gene tagging has evolved from a methods-development effort into a routine enabling platform for cell biology, disease modeling, and drug discovery. These advances include a mechanistically understood balance between NHEJ and HDR repair pathways, an expanded menu of protein tags that span fluorescent, luminescent, affinity, epitope, and proximity-labeling readouts, well-defined design principles for sgRNA, donor, and tag placement, and the multiplex tagging of up to three independent genes in the same cell [101]. The dividends of this maturation are already evident in large-scale tagging atlases such as OpenCell and the Allen Cell Collection, isogenic iPSC-derived disease models following tagged proteins through differentiation, and high-throughput drug-discovery platforms quantifying target engagement, protein degradation, and compound mechanism of action at native expression. Endogenous tagging should therefore not be considered a technique but rather an enabling infrastructure that connects genome engineering with live-cell imaging, proteomics, and translational pharmacology.

However, there are still several bottlenecks to be overcome. Current strategies cannot fully overcome the cell-cycle restriction of the pathway, and post-mitotic and primary cells still exhibit low HDR efficiency. Multiplex tagging suffers from compounding efficiency loss as the number of loci increases, which limits how far the parallel-tagging concept can be pushed in a single clonal line. Double-strand breaks induced by CRISPR/Cas9 remain cytotoxic in iPSCs and drive karyotype drift and clonal heterogeneity [143].

Two technological developments are likely to shape the next phase in the field. The first and arguably most impactful is the arrival of DSB-free CRISPR knock-in approaches. Prime editing uses a Cas9 nickase fused to a reverse transcriptase, guided by a pegRNA to introduce point mutations, short insertions, and short deletions in human cells without the need for a double strand break or exogenous donor [134]. PASTE technology embodies this logic for large-cargo integration by combining a serine integrase with a prime editor: the prime editor installs a short attB landing pad at the target locus, and the integrase then delivers cargos of up to ~36 kb at 10–50% efficiency in human cells and mouse liver, again without generating a DSB [139]. Alternatively, CRISPR-associated transposase systems can be employed. In addition, the laboratory-evolved Type I-F variant evoCAST mediates 10–30% targeted insertion of kilobase-scale cargo at fourteen human genomic loci with undetectable indels and low off-target integration [142]. Taken together, these new methods hold great promise for the programmable insertion of fluorescent, luminescent, and epitope tags into post-mitotic neurons, hepatocytes, and primary immune cells where HDR is still very challenging. The second is the extension of the endogenous tagging into intact tissues and whole-organism models instead of just cultured cells, which will enable the same physiological readouts that now define cell-culture pharmacology to be deployed in animal models. These advances are expected to transform the field in the next few years from cataloguing tagged cell lines to functional, quantitative cell biology and translational pharmacology at endogenous expression.

## Data Availability

No new data were created or analyzed in this study. Data sharing is not applicable to this article.
